# Racial and Ethnic Disparities Among Participants in US-Based Phase 3 Randomized Cancer Clinical Trials

**DOI:** 10.1093/jncics/pkaa060

**Published:** 2020-07-07

**Authors:** Stephen R Grant, Timothy A Lin, Austin B Miller, Walker Mainwaring, Andres F Espinoza, Amit Jethanandani, Gary V Walker, Benjamin D Smith, B Ashleigh Guadagnolo, Reshma Jagsi, C David Fuller, Charles R Thomas, Ethan B Ludmir

**Affiliations:** p1 The University of Texas MD Anderson Cancer Center, Houston, TX, USA; p2 The Johns Hopkins University School of Medicine, Baltimore, MD, USA; p3 The University of Texas Health Science Center McGovern Medical School, Houston, TX, USA; p4 Baylor College of Medicine, Houston, TX, USA; p5 The University of Tennessee Health Science Center College of Medicine, Memphis, TN, USA; p6 Banner MD Anderson Cancer Center, Gilbert, AZ, USA; p7 The University of Michigan, Ann Arbor, MI, USA; p8 Oregon Health & Science University, Portland, OR, USA

## Abstract

Although improving representation of racial and ethnic groups in United States clinical trials has been a focus of federal initiatives for nearly 3 decades, the status of racial and ethnic minority enrollment on cancer trials is largely unknown. We used a broad collection of phase 3 cancer trials derived from ClinicalTrials.gov to evaluate racial and ethnic enrollment among US cancer trials. The difference in incidence by race and ethnicity was the median absolute difference between trial and corresponding Surveillance, Epidemiology, and End Results data. All statistical tests were 2-sided. Using a cohort of 168 eligible trials, median difference in incidence by race and ethnicity was +6.8% for Whites (interquartile range [IQR] = +1.8% to +10.1%; *P* < .001 by Wilcoxon signed-rank test comparing median difference in incidence by race and ethnicity to a value of 0), -2.6% for Blacks (IQR = -5.1% to +1.2%; *P* = .004), -4.7% for Hispanics (IQR = -7.5% to -0.3%; *P* < .001), and -4.7% for Asians (IQR = -5.7% to -3.3%; *P* < .001). These data demonstrate overrepresentation of Whites, with continued underrepresentation of racial and ethnic minority subgroups.

In 1993, the National Institute of Health (NIH) Revitalization Act (1–3) detailed a plan for the inclusion of racial and ethnic minority groups in clinical research in the United States. Numerous studies have since examined racial and ethnic minority representation on cancer trials, with the large majority demonstrating overrepresentation of Whites with underrepresentation of racial and ethnic minorities (4–6). There is limited data, however, on recent enrollment trends in US-only cancer trials, few reports on Native American and Hawaiian groups, and little known of the association between racial and ethnic enrollment and trial type. To that end, we used a broad clinical trials database to determine racial and ethnic enrollment disparities in recent US clinical trials with the hypothesis that historically underrepresented minorities will be underenrolled on recent cancer clinical trials.

ClinicalTrials.gov was queried by the following search parameters: terms: “cancer”; study type: “all studies”; status: excluded “not yet recruiting”; phase: phase 3; and study results: “with results.” Of 1239 identified trials, 168 addressed a therapeutic intervention with exclusive US enrollment. The ClinicalTrials.gov database and manuscript publications were reviewed for race and ethnicity reporting. Race and ethnicity were self-reported, with exclusion of multiple or unknown race. All current US cooperative groups (Southwest Oncology Group, Eastern Cooperative Oncology Group–American College of Radiology Imaging Network, NRG Oncology, Alliance, and Children’s Oncology Group) were represented in this dataset. Incidence of race and ethnicity in the US cancer population was estimated using the “Race recode” and “Origin recode” of the Surveillance, Epidemiology, and End Results (SEER) database, extracted in 5-year increments with race and ethnicity considered separately.

Trial and SEER data were compared, correlating with the median year of patient enrollment. Comparative SEER data was filtered for the disease site of interest, except for trials where enrollment included patients with more than 3 primary disease sites. The difference in incidence by race and ethnicity (D-IRE) was defined as the median absolute difference in race and ethnicity incidence between trial and corresponding disease-specific SEER data, with a negative value indicating underrepresentation (Table 1). The ratio of incidence by race and ethnicity (R-IRE) was defined as the median ratio of trial (numerator) and SEER (denominator) incidence, with a value less than 1 indicating underrepresentation. D-IRE and R-IRE values for each race and ethnicity were calculated only for trials that reported on that particular subgroup. Statistical tests were nonparametric with an a priori threshold of α = 0.05 for statistical significance. Mann-Whitney U and Kruskall-Wallis analysis of variance (ANOVA) were used to compare subgroups. The Wilcoxon signed-rank test was used to compare median D-IRE for each race and ethnicity to a value of 0 and R-IRE to a value of 1 (null hypotheses).

**Table:** Table 1. Difference in incidence by race/ethnicity (%)

	No. trials	White			Black			Hispanic			Asian		
		Median (IQR)		*P* ^a^	Median (IQR)		*P* ^a^	Median (IQR)		*P* ^a^	Median (IQR)		*P* ^a^
All included trials	96	+6.8 (+1.8 to +10.1)		<.001	−2.6 (-5.1 to +1.2)		.004	−4.7 (-7.5 to -0.3)		<.001	−4.7 (-5.7 to -3.3)		<.001
Median year													
<2005	15	+6.5 (+3.2 to +10.4)		.93	−2.7 (-4.6 to +1.7)		.58	−1.5 (-5.0 to +0.4)		.76	−5.3 (-5.7 to -3.7)		.96
2005-2007	26	+6.3 (+3.7 to +11.1)			−3.4 (-7.5 to -0.3)			−5.3 (-7.9 to -0.9)			−4.5 (-5.6 to -3.8)		
2008- 2010	35	+6.1 (+1.5 to +9.6)			−2.1 (-4.2 to +1.1)			−4.7 (-7.4 to -0.4)			−4.7 (-5.8 to -2.3)		
>2010	20	+7.6 (+0.4 to +10.1)			−2.6 (-5.0 to +2.0)			−4.6 (-9.2 to -2.5)			−4.6 (-5.7 to -3.3)		
Cooperative group													
No	34	+5.0 (-0.5 to +11.0)		.25	−1.4 (-3.2 to +1.8)		.24	−1.8 (-6.7 to +0.4)		.23	−4.7 (-5.7 to -2.2)		.48
Yes	62	+7.8 (+3.0 to +10.0)			−3.1 (-5.7 to +0.5)			−4.9 (-7.9 to -2.8)			−4.8 (-5.7 to -3.5)		
Industry funded													
No	60	+7.6 (+1.7 to +9.9)		.89	−2.7 (-6.1 to +0.9)		.69	−5.2 (-8.1 to -0.4)		.16	−4.9 (-5.7 to -3.3)		.67
Yes	36	+6.3 (+1.8 to +10.4)			−2.3 (-4.6 to +1.6)			−3.4 (-4.7 to -0.3)			−4.5 (-5.8 to -3.3)		
Disease site													
Breast	18	+8.0 (+1.8 to +10.4)		.35	−2.7 (-6.7 to +1.7)		.38	−4.7 (-9.2 to -3.4)		.26	−5.5 (-7.1 to -3.4)		.27
Colorectal	5	+7.7 (+6.2 to +11.5)			−4.1 (-6.8 to -3.0)			+4.3 (-9.2 to -3.4)			−5.1 (-6.7 to -4.1)		
Lung	5	+3.3 (+1.5 to +5.5)			−1.7 (-3.0 to +1.7)			−4.7 (-5.9 to -3.4)			−3.1 (-4.5 to -2.2)		
Prostate	8	+3.1 (-0.2 to +8.0)			+1.6 (-4.2 to +5.1)			−4.4 (-5.2 to +0.0)			−4.2 (-4.7 to -2.6)		
Primary modality													
Supportive care	44	+8.0 (+1.8 to +10.1)		.10	−3.9 (-6.7 to -1.3)		.09	−6.5 (-8.8 to -1.7)		.19	−5.0 (-5.8 to -3.4)		.56
Targeted systemic therapy	26	+8.8 (+1.9 to +11.5)			−2.7 (-5.2 to +1.3)			−4.1 (-5.0 to +0.0)			−4.4 (-5.5 to -3.8)		
Cytotoxic chemotherapy	19	+3.4 (+1.8 to +10.0)			+0.1 (-3.0 to +6.1)			−2.7 (-4.3 to +0.1)			−3.8 (-5.7 to -2.2)		
Radiation or surgery	7	+4.0 (-2.3 to +6.7)			−2.2 (-4.6 to +2.0)			−6.3 (-9.2 to +10.3)			−4.4 (-5.7 to +1.2)		

a Median differences in incidence by race and ethnicity (D-IRE) for each group with IQR are displayed. *P* values for the top line (all included trials without further subgrouping) were calculated by a Wilcoxon signed-rank test comparing median D-IRE to a value of 0 (null hypothesis). All other *P* values were calculated by Mann Whitney U (for variables with 2 subgroups) or Kruskall-Wallis ANOVA (for variables with more than 2 subgroups). ANOVA = analysis of variance; IQR = interquartile range.

Of 168 eligible trials, 96 (57.1%) reported the proportion of at least 1 race and ethnicity, representing 34 329 patients. Of these 96 trials, 97.9% reported a proportion of White enrollees compared with 84.4% reporting Black, 52.1% Hispanic, 67.7% Asian, 61.4% Native American, and 56.3% Native Hawaiian and Pacific Islander. The median proportion of White enrollees on the included trials was 88.7% compared with 8.6% Black, 4.0% Hispanic, 1.4% Asian, 0.1% Native American, and 0.0% Native Hawaiian and Pacific Islander. Cooperative group–sponsored trials were more likely to report race and ethnicity (66.0% vs 45.9% for noncooperative group–supported trials; *P* = .02).

The median D-IRE was +6.8% for Whites (interquartile range [IQR] = +1.8% to +10.1%; *P* < .001 by Wilcoxon signed-rank test comparing median D-IRE to a value of 0), −2.6% for Blacks (IQR = −5.1% to +1.2%; *P* = .004), −4.7% for Hispanics (IQR = −7.5% to −0.3%; *P* < .001), and −4.7% for Asians (IQR = −5.7% to −3.3%; *P* < .001) (Table 1 and Figure 1). The median R-IRE was 1.08 for Whites (IQR = 1.02-1.12; *P* < .001), 0.76 for Blacks (IQR = 0.47-1.09; *P* = .003), 0.50 for Hispanics (IQR = 0.16-0.96; *P* < .001), and 0.19 for Asians (IQR = 0.08-0.45; *P* < .001) (Figure 1). There was no difference in the D-IRE over time (median trial enrollment date <2005 vs 2005-2007 vs 2008-2010 vs >2010) for any race and ethnicity. A sensitivity analysis excluding trials enrolling multiple disease sites (22 of 96 trials) showed similar findings by racial and ethnic groups (D-IRE of +6.3% for Whites [IQR = +1.8% to +10.2%; *P* < .001], −2.5% for Blacks [IQR = −4.6% to +1.5%; *P* = .03], −4.5% for Hispanics [IQR = −6.8% to −0.4%; *P* = .001], and −4.6% for Asians [IQR = −5.8% to −3.1%; *P* < .001]).

**Figure 1.**
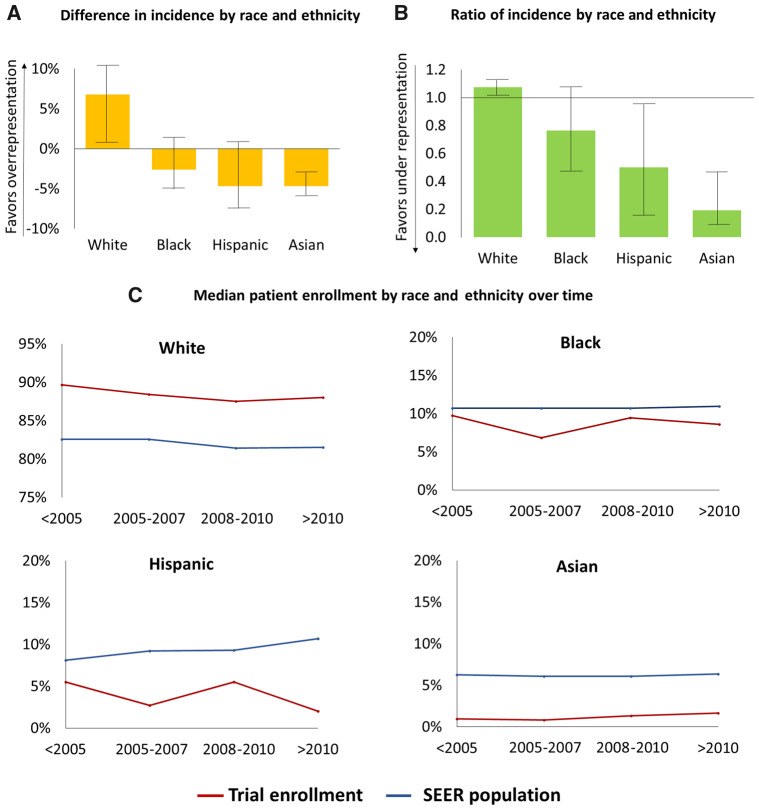
Median difference in incidence by race and ethnicity with interquartile range shown with error bars (**A**), median ratio of incidence by race and ethnicity with interquartile range shown with error bars (**B**), and median proportions of race and ethnicity compared with Surveillance, Epidemiology, and End Results (SEER) estimates (**C**).

Equitable representation on US cancer clinical trials is necessary to ensure generalizable results and allow for equal access to new treatment advances and has been an explicit priority for NIH-supported clinical trials for nearly 3 decades (1–3). Potential drivers of underrepresentation are complex and include narrow eligibility criteria, lack of access to participating centers, patient preference, and fear and/or mistrust, as well as socioeconomic, language, and cultural factors (7–9). Seminal work published more than 15 years ago examining NIH cooperative group trials demonstrated similar underrepresentation of racial and ethnic minority subgroups (4). Further work is thus needed to identify and address continued barriers of enrollment faced by minority groups and to more fully understand the impact of NIH efforts on their enrollment.

The primary limitation of this study is the large number of trials (43%) not reporting any race or ethnicity and inconsistent race and ethnicity groupings across studies and the associated bias this may introduce. Results should thus be interpreted with caution. In addition, although other covariates such as age, sex, and socioeconomic factors are known to correlate with race and ethnicity disparities (10,11), these could not be fully evaluated given use of aggregate race data. Ongoing studies with granular patient-level data are thus needed to elucidate specific underrepresented patient populations. There are also limitations with the comparator SEER dataset, which, although largely representative of the general US population, captures only 25%-28% of incident cancer cases nationwide and may underrepresent Black and Hispanic patients (12). Nonetheless, to our knowledge, this analysis represents the index aggregate utilization of federal public access datasets to assess domestic disparities in racial and ethnic phase 3 clinical trial enrollment. Although others have investigated Food and Drug Administration–approval trials (13), our use of US-limited, large-scale public access datasets affords a benchmark for future serial assessments and US policy decisions.

In conclusion, more than 40% of US cancer clinical trials fail to report race or ethnicity data. Of reporting trials, White patients continue to be overrepresented, whereas racial and ethnic minority subgroups are underrepresented. Mandatory, standard, and granular reporting of racial and ethnic data elements should be considered in future iterations of ClinicalTrials.gov and other US cancer datasets, with continued efforts needed to ensure equitable clinical trial enrollment.

## Funding

The authors report no funding for this work.

## Notes

**Disclosures:** The authors report no financial disclosures or conflicts of interests related to this work.

**Author contributions**: SRG: Conception and design; Data acquisition, analysis, and interpretation; Drafted initial manuscript; Drafting, revising, editing manuscript. TAL: Data acquisition, analysis, and interpretation; Drafting, revising, editing manuscript. ABM: Data acquisition, analysis, and interpretation; Drafting, revising, editing manuscript. WM: Data acquisition, analysis, and interpretation; Drafting, revising, editing manuscript. AFE: Data acquisition, analysis, and interpretation; Drafting, revising, editing manuscript. AJ: Data acquisition, analysis, and interpretation; Drafting, revising, editing manuscript. GVW: Drafting, revising, editing manuscript. BDS: Drafting, revising, editing manuscript. BAG: Drafting, revising, editing manuscript. RJ: Drafting, revising, editing manuscript. CDF: Conception and design; Drafting, revising, editing manuscript. CRT: Conception and design; Drafting, revising, editing manuscript. EBL: Conception and design; Data acquisition, analysis, and interpretation; Editorial oversight and correspondence; Drafting, revising, editing manuscript.

## Data availability

All available data can be obtained by contacting the corresponding author.
